# A Low-Power General Matrix Multiplication Accelerator with Sparse Weight-and-Output Stationary Dataflow

**DOI:** 10.3390/mi16010101

**Published:** 2025-01-16

**Authors:** Peng Liu, Yu Wang

**Affiliations:** Research Center for Novel Computing Sensing and Intelligent Processing, Zhejiang Lab, Hangzhou 311100, China; zationlue@tju.edu.cn

**Keywords:** GEMM accelerator, dataflow, sparsity

## Abstract

General matrix multiplication (GEMM) in machine learning involves massive computation and data movement, which restricts its deployment on resource-constrained devices. Although data reuse can reduce data movement during GEMM processing, current approaches fail to fully exploit its potential. This work introduces a sparse GEMM accelerator with a weight-and-output stationary (WOS) dataflow and a distributed buffer architecture. It processes GEMM in a compressed format and eliminates on-chip transfers of both weights and partial sums. Furthermore, to map the compressed GEMM of various sizes onto the accelerator, an adaptable mapping scheme is designed. However, the irregular sparsity of weight matrices makes it difficult to store them in local buffers with the compressed format; denser vectors can exceed the buffer capacity, while sparser vectors may lead to the underutilization of buffers. To address this complication, this work also proposes an offline sparsity-aware shuffle strategy for weights, which balances the utilization of distributed buffers and minimizes buffer waste. Finally, a low-cost sparse computing method is applied to the WOS dataflow with globally shared inputs to achieve high computing throughput. Experiments with an FPGA show that the proposed accelerator achieves 1.73× better computing efficiency and 1.36× higher energy efficiency than existing approaches.

## 1. Introduction

**Figure 1:**
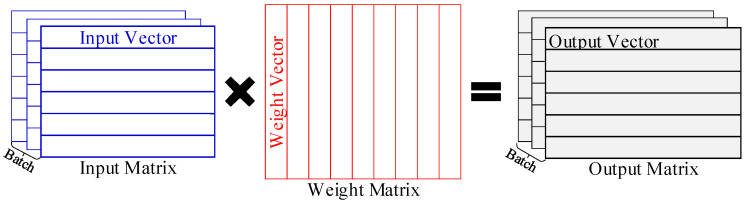
GEMM operations.

General matrix multiplication (GEMM) is an indispensable computing pattern for most deep learning models, mainly used for multiplying input matrices with weight matrices [1,2], as shown in Figure 1. Thus, it is essential for deep learning platforms, such as GPUs and TPUs, to accelerate GEMM efficiently [3]. However, these platforms consume significant energy, making them unsuitable for resource-constrained applications [4,5]. Meanwhile, the matrices currently used in deep learning, including convolutional neural networks (CNNs), recurrent neural networks (RNNs), and transformer-based neural networks, are usually very large in size [6,7,8,9]. This results in considerable data movement and massive computation during GEMM processing, creating a bottleneck for deploying GEMM in resource-constrained applications [10,11].

DRAM accesses and on-chip transfers consume much more energy than computing and accessing small-size on-chip buffers [12]. Therefore, various dataflows have been proposed for GEMM accelerators to reduce DRAM accesses and on-chip transfers, including output-stationary (OS), weight-stationary (WS), and row-stationary (RS) dataflows [12,13,14]. WS [15] and OS [16] dataflows fix one type of data—either weights or partial sums—in processing elements (PEs) for full reuse during computation. However, they need to repetitively access and transfer all other data, which means the accelerators are still energy-intensive [12]. Although row-stationary (RS) dataflow can maintain multiple types of data in PEs, it requires a complicated buffer architecture and a costly network-on-chip (NoC), incurring notable overhead [14]. Thus, a multiple-data-stationary dataflow with minimal overhead is required for resource-constrained applications. At the same time, most GEMM accelerators use a centralized buffer architecture, which suffers from low flexibility, limited bandwidth, and inadequate data reuse, making it unsuitable for multiple dataflows [17]. In contrast, a few studies have adopted a distributed buffer architecture for greater flexibility, such as works [18,19]. This architecture can be configured to support various dataflows. Unfortunately, it also leads to data duplication on-chip [17] and needs to store the same data repeatedly in several distributed buffers. This practice reduces the utilization of buffers and causes additional energy consumption [17].

Meanwhile, both input and weight matrices in machine learning are very sparse, which means that they contain many zeros [20,21]. Therefore, they can be compressed into a much smaller size to save on storage and access overhead [22,23,24]. Moreover, since zeros do not contribute to the multiply–accumulate (MAC) computation, they can be skipped to reduce computing latency or powered off to save energy [25,26,27]. Many works have studied the exploitation of sparsity in neural network accelerators, but few have combined sparse processing with multiple-data-stationary dataflow, which is not naturally suitable for fully compressed GEMM [28]. This creates additional challenges for the accelerator, especially when integrated with the distributed buffer architecture.

First, GEMM operations have varied dimensions, and the actual dimensions of compressed matrices are unpredictable [2,29]. Thus, it is impossible to deploy GEMM onto the accelerator using a fixed mapping scheme [30]. Even GEMM operations with the same original dimensions may require different mapping schemes after compression [31,32]. Inefficient mapping could lead to the significant under-utilization of PEs. Therefore, it is necessary to design a flexible mapping scheme that adaptively considers both the original and compressed dimensions of GEMM operations.

Secondly, vectors in a compressed matrix have diverse lengths due to their irregular sparsity ratio, resulting in an irregular shape for the compressed matrix [5,33]. When stored in distributed buffers, these vectors may exceed buffer capacity or fail to utilize it efficiently [34]. This can lead to significantly imbalanced utilization among buffers and waste storage space [35]. Therefore, the storage policies for compressed GEMM must consider the sparsity of vectors and reorder them into a balanced format before loading them into the on-chip buffers.

Finally, many works exploit the sparsity in the computation to reduce both latency and energy consumption by processing GEMM in the compressed format [33,36]. However, some works leverage the sparsity in only one operand of the GEMM, either the inputs or weights. For example, NullHop [19] only compresses the inputs and stores the weights in the original format. Some works process both inputs and weights in the compressed format, but this induces significant overhead in hardware resources and energy consumption [37]. In resource-constrained applications, it is important to achieve high efficiency in resource utilization on the accelerator. Current works either fail to fully leverage the sparsity of GEMM to improve the efficiency or over-exploit it, incurring high overhead [38].

To address the above issues, this paper proposes a low-power sparse GEMM accelerator for resource-constrained applications. First, the weight-and-output stationary (WOS) dataflow is designed with the distributed buffer architecture. Each weight vector remains stationary in one buffer and the computation of each output is fixed in one PE, eliminating the on-chip transfers of both weights and partial sums. Additionally, an adaptable mapping scheme (AMS) is proposed for the WOS dataflow to accommodate compressed GEMM of varying sizes while maintaining high PE utilization. Second, a sparsity-aware shuffle strategy is designed to reorder weight vectors offline based on their sparsity. The sparser vectors will be complemented with denser ones in both vector and block levels logically to evenly utilize the distributed buffers. A physical buffer design is also presented to implement the offline logical storage schedule. Third, a sparse computing method is designed based on WOS dataflow with low overhead. It processes both inputs and weights in the compressed format, thereby skipping the computation with zeros in the inputs to reduce the latency and powering off the PEs that compute zeros in the weights to save energy. All transfer and storage of zeros is also eliminated. Finally, the proposed GEMM accelerator is implemented and validated on FPGA. Experiments show that the proposed accelerator achieves 1.73× better computing efficiency and 1.36× higher energy efficiency than existing works. The main contributions are listed as follows:

- The WOS dataflow is proposed for the GEMM operations, and a novel accelerator is designed based on it with a distributed buffer architecture. An adaptable mapping scheme is also presented to accommodate compressed GEMMs of varying sizes;
- An offline sparsity-aware shuffle strategy is presented to reorder the weight vectors into a matrix with regular shape logically, using a two-level complementary schedule. A novel physical buffer design is also adopted accordingly;
- A low-cost sparse processing method is introduced. It globally shares the indices of compressed inputs and locates the corresponding weights in each local buffer using indirect addressing. The sparse GEMM accelerator is implemented with FPGA and tested with GEMM operations from various neural networks to verify its efficiency.

The rest of the paper is organized as follows. Section 2 introduces the preliminaries of this paper. Section 3 presents the architecture and dataflow of the proposed accelerator. Then, the sparse and balanced storage system is described in Section 4, while Section 5 demonstrates the sparse computing method and flow. Section 6 details the implementation, experiments, and discussions. Finally, the paper concludes in Section 7.

## 2. Preliminaries

### 2.1. The GEMM Operations

General matrix multiplication (GEMM) is one of the primary operations in machine learning [1]. It involves multiplying two matrices to generate a third matrix [4]. GEMM is widely used in different components of various neural networks, such as the multilayer perceptron (MLP), the fully-connected (FC) layers in CNNs, the gating mechanisms in RNNs and the attention mechanisms in transformers [39,40,41]. Moreover, other operations, such as convolution layers, can also be converted into GEMM. Therefore, efficiently accelerating GEMM operations is essential in modern artificial intelligence applications.

In machine learning, most GEMM operations involve multiplying the input matrix with the weight matrix to generate the output matrix. These operations are typically both computation- and memory-intensive, involving vast amounts of computation and data. For example, in CNNs, an FC layer may contain millions of MAC operations [10,14]. As a result, processing GEMM operations on hardware platforms can be resource-intensive, consuming significant hardware resources and energy, while also incurring high latency. This creates a severe bottleneck in machine learning applications, especially in the resource-constrained environments, such as embedded, mobile, or edge devices. Furthermore, GEMM often involves very large weight matrices. There are 65 million weights in the transformer [42]. In the CNNs, weights in FC layers can account for more than 90% of total network size [43]. The extensive storage and access of weights poses severe challenges, necessitating large on-chip buffers, costly NoCs and high-bandwidth DRAM [23,31]. Thus, an efficient GEMM accelerator should maximize the reuse of all data, especially the weights.

### 2.2. The Dataflows-Based Accelerators

Dataflow defines the computing flow, data reuse patterns and mapping schemes in the accelerator. Several dataflows have been proposed in earlier works, including the weight-stationary (WS) dataflow, the output-stationary (OS) dataflow, the row-stationary (RS) dataflow and no-local-reuse (NLR) dataflow [12,13,14]. Compared with NLR dataflow, WS, OS and RS dataflows have shown high efficiency in neural network accelerators by keeping certain data stationary in the PEs to increase the on-chip data reuse [12,17]. This reduces the amount of data transfers on-chip, leading to notable energy savings.

Many accelerators have been proposed based on various dataflows for higher efficiency. However, WS and OS dataflow accelerators only keep weights and outputs in the Pes, respectively, while all other data still need to be transferred [17]. On the other hand, since the number of weights is very large in GEMM operations, it is important to improve the reuse of weights. Fixing the weights in PEs is one way to achieve higher reuse. However, a multiple-data-stationary dataflow with minimal overhead will be more efficient [12,17]. RS dataflow accelerators, on the other hand, retain more types of data in the PEs, but require a more complex NoC and buffer architecture, resulting in considerable overhead in both hardware and energy consumption. Similar issues also arise in heterogeneous or reconfigurable dataflow architectures. Fortunately, the distributed buffer architecture has shown potential for combining multiple dataflows. Meanwhile, the sparsity in the data can be exploited to shrink the size of GEMM, alleviating the stress induced by the storage, transfer, and access of data.

### 2.3. The Exploitation of Sparsity in the GEMM

The matrices in GEMM are usually very large, resulting in massive computation, data transfer and storage [22,23,24,25]. On the other hand, GEMM operations are highly sparse, with both the input matrices and weight matrices containing many zeros. Therefore, both the input matrices and output matrices can be compressed into smaller sizes, significantly reducing the overhead of storage and the access from DRAM [21]. Moreover, when processing the compressed GEMM without decompression, the on-chip transfer of zeros is eliminated, further reducing energy consumption [26]. Additionally, skipping computations with zeros can reduce the processing latency, which brings about higher computing throughput [24]. When PEs are computing zeros, they can also be powered off to save energy [16].

Currently, many works have discussed the sparse processing methods [31,32,33,34,35,36,37]. However, few of them are designed for the WOS dataflow and distributed buffer architecture [33]. For example, they cannot address the imbalanced utilization of local buffers caused by the varied lengths of compressed data [36]. Although the sparse accelerator, NullHop [19], adopts the WOS dataflow and distributed buffer architecture, it is specifically designed for convolutional layers in CNNs. Moreover, it only exploits the sparsity in inputs with compressed format, while zeros in weights are still stored, transferred, and computed. Besides this, existing sparse processing methods usually cause non-negligible overhead [38]. Thus, a low-cost sparse processing method for the WOS dataflow accelerator based on the distributed architecture warrants further investigation.

### 2.4. Related Works of GEMM Accelerators

Many works have proposed GEMM accelerators, but only a few of them have discussed the dataflows within them. Most of these accelerators only support one dataflow, which cannot fully exploit data reuse. Works [9,13,14,17] are designed for multiple dataflows. However, they adopt the heterogeneous, reconfigurable or flexible architecture, requiring extensive hardware and energy overhead. Works [12,33] utilize the distributed buffer architecture to achieve high adaptability for dataflows, but they suffer from repetitive on-chip storage. Since most matrices contain many zeros, some GEMM accelerators have tried to leverage the sparsity to reduce the processing latency and energy. Works [25,32,34] only exploit the sparsity in one side of the GEMM, either the weights or inputs. They store and process the other side with an uncompressed format. Works [20,24] compress both sides of GEMM, but they incur large overhead to skip the computation involving zeros from both sides. Works [4,44] try to reduce the overhead, but they are only applicable to structured sparsity.

## 3. The Architecture and Dataflow of Proposed GEMM Accelerator

### 3.1. The Primary Architecture

**Figure 2:**
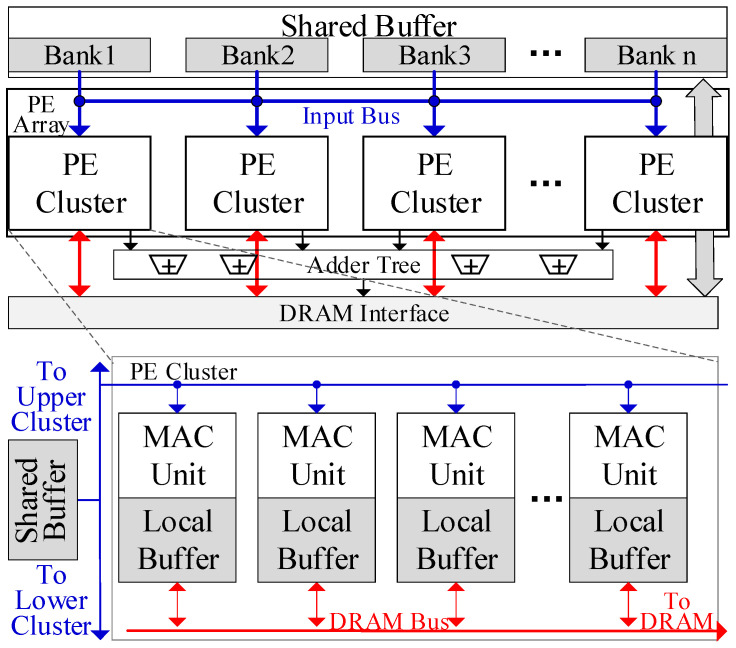
Architecture of the proposed GEMM accelerator.

Figure 2 shows the architecture of the proposed GEMM accelerator. The PE array is the basic computing unit, with each PE comprising several multiply–accumulate (MAC) units and registers. Each PE is paired with an exclusive local buffer that stores weights. Multiple PEs are connected in a 1-D chain to form a PE cluster, where data can be transferred between adjacent PEs within the cluster. Each PE cluster is connected to a shared buffer bank, which stores the inputs. Inputs in the shared buffer bank can be broadcast in a systolic manner to all PEs within the cluster for full reuse.

The PE array is composed of several PE clusters, which are also connected in a 1-D chain. Adjacent PE clusters can share the inputs and all shared buffer banks can function as a unified buffer. Hence, inputs can be reused across the entire PE array. The PE array can be divided into several PE groups, each containing multiple adjacent PE clusters. PE clusters within the same group share the inputs and computing flow, and their shared buffer banks function as a single unified buffer. An adder tree is connected to the PE Array for the further processing of the outputs of PEs, as presented in the adaptable mapping scheme.

Each PE cluster can independently access the DRAM interface to load weights or transfer the outputs to the DRAM. In each cluster, only one PE can communicate with the DRAM bus at a time. Each PE will access the DRAM sequentially. This design can simplify the NoC to save on resource consumption. Auxiliary modules are also equipped on-chip, including the controller and the compression unit.

### 3.2. Weight-and-Output Stationary Dafaflow

**Figure 3:**
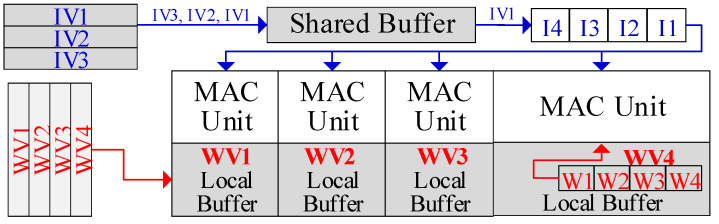
WOS dataflow. IV and WV refer to input and weight vectors, while I1-4 and W1-4 denote the inputs and weights, respectively.

The proposed WOS dataflow is illustrated with a single PE cluster for brevity, as shown in Figure 3. First, each PE’s local buffer holds a distinct weight vector. The input vectors are stored in the shared buffer bank. Each PE is responsible for computing its stored weight vector with all the input vectors to produce the outputs.

The input vectors are computed sequentially. First, the shared buffer will broadcast an input in a vector to all PEs. Then, each PE can obtain the corresponding weight from its local buffer and perform MAC computation with the received input. Next, all the inputs in the vector will be broadcast and computed sequentially. Each PE will hold the partial sum in the register. Once all the inputs in the vector are processed, each PE produces an output. Accordingly, the PE cluster will produce a row (or a portion of the row) in the output matrix. Afterward, the next input vector is broadcasted and computed to generate another row of outputs. After processing the entire input matrix, each PE contributes a column to the output matrix, and the cluster ultimately produces the output matrix. Then, another input matrix in the batch can be loaded into the shared buffer and computed, while the weight vectors remain in the local buffer. Finally, all input matrices are computed and the weights vectors are exhaustively used. The above operations can be generalized to a PE group, ranging from one cluster to the entire PE array.

WOS dataflow can maximize data reuse in GEMM. We assume the input matrix has I vectors and the batch size is B. During the computation of each batch, weight vectors are reused for I×B iterations. All partial sums also stay in each PE. No transfers happen to either the weights or the partial sums. Meanwhile, all inputs are shared by all PEs. Moreover, each PE can hold multiple weight vectors in its local buffer, and inputs can be computed with all of them. Both weights and inputs will be fully reused once loaded into buffers, and partial sums will not be stored into DRAM, thus minimizing repetitive DRAM accesses for the same data. In this way, WOS dataflow maximizes the reuse of all data, including inputs, weights and partial sums. This not only significantly reduces the on-chip data transfers, but also effectively cuts down the DRAM accesses.

It should be noted that all the data will remain compressed both on- and off-chip to save energy and memory spaces. The inputs and outputs are compressed using modified Run Length Encoding (RLE), similar to the encoding method in EIE [20]. In this format, only the nonzero data are stored, each paired with a code indicating the number of zeros between it and the preceding nonzero data. This format is chosen because it is computation-friendly. Its code can be globally shared to all PEs as index directly; a 4-bit code is adopted in this paper, the same as EIE [20].

For the weight vectors, only nonzero weights are stored in the compressed format. Each weight is paired with a bit flag; it is set to 0 if the weight is zero, and to 1 otherwise. A bitmap is added to each local buffer to store these bit flags. This compressed format is space-efficient with low overhead, which is crucial for the WOS dataflow, as it is used in each PE.

### 3.3. Adaptable Mapping Scheme (AMS) for WOS Dataflow

The GEMM operations should be mapped onto the accelerator before processing them. It should try to fully utilize PEs in the accelerator for computation, which will improve the actual computing throughput of the accelerator. Meanwhile, all required data should be stored on-chip and easily accessed for computing. The size of GEMM operations varies greatly among different applications. The number of weight vectors may not always match the number of PEs in the accelerator, potentially causing the under-utilization of PEs. Also, the length of a vector may exceed the local buffer capacity. More importantly, the lengths of compressed vectors vary greatly and are very irregular. Thus, it is not feasible to map different compressed GEMM operations onto the accelerator in the same way, even though they may share the same original GEMM. It is essential to exploit the fine-grained mapping scheme to maximize PE utilization.

To address this issue, the adaptable mapping scheme (AMS) is proposed for WOS dataflow, which can accommodate GEMM operations of various sizes while ensuring high PE utilization. We assume the PE array of the accelerator contains N PE clusters, each containing P PEs.

#### 3.3.1. Primary Mapping Method

When mapping the GEMM on the PE array, its weight matrix is loaded into the PEs first. Regularly, the weight matrix is divided into blocks (or submatrices), each containing N×P  vectors. Then, we load each block into the accelerator and compute it with all input vectors in the WOS dataflow.

The PE array can be divided into PE groups, as mentioned in Section 3.1. This configuration enhances the accelerator’s flexibility in size. However, a weight block may contain fewer than N×P vectors, which will leave some PEs unused. More importantly, the irregular lengths of compressed vectors may not align with the fixed capacity of local buffers, leading to unpredictable buffer utilization. The primary mapping method cannot handle these two irregular cases. Therefore, the specific mapping methods are implemented based on the actual dimensions of the compressed weight matrices.

#### 3.3.2. Mapping Method for Case 1

**Figure 4:**
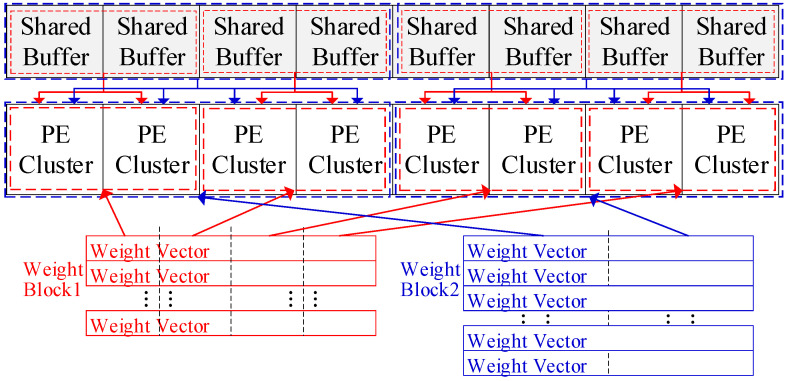
Divide 2 weight blocks and map them with 8 PE clusters. The red block is divided into 4 subblocks while the blue block is divided into 2 subblocks.

As discussed above, the weight matrix is divided into blocks prior to mapping. We assume a weight block contains L weight vectors. If L<NP, this block will leave some PEs unused in the accelerator. To address this issue, first, if L≤NP/2, the coarse-grained mapping scheme is applied to the WOS dataflow. Each weight vector in the uncompressed format will be evenly split into $\left\lfloor N/\left\lceil L/P\right\rceil \right\rfloor$ segments, resulting in the division of the block into $\left\lfloor N/\left\lceil L/P\right\rceil \right\rfloor$ subblocks. The input vectors are divided into $\left\lfloor N/\left\lceil L/P\right\rceil \right\rfloor$ segments correspondingly. The PE array is also split into $\left\lfloor N/\left\lceil L/P\right\rceil \right\rfloor$ groups, with each group containing $\left\lceil L/P\right\rceil$ PE clusters. Fewer than $\left\lceil L/P\right\rceil$ PE clusters may remain unused and unassigned to any group. We load each subblock into a PE group, and its corresponding input segments are loaded into the shared buffer. Each PE will store a segment of the weight vector and compute it with inputs. The outputs of PEs with the same position in each group will be added together in an adder tree to produce the final outputs. Figure 4 shows two examples of the division and mapping of weight blocks.

More aggressively, at the fine-grained level, if $NP/2<L\leq \left\lfloor N/3\right\rfloor P+NP/2$, this block can first be divided into two smaller blocks: one containing NP/2 vectors and the other containing the remaining vectors. Then, two smaller blocks can be processed as irregular blocks, as described in the above paragraph. If $L>\left\lfloor N/3\right\rfloor P+NP/2$, the primary mapping method and the mapping method for Case 1 will result in the same PE utilization for this block, which is relatively high. Meanwhile, the latter method will incur slight extra energy overhead for transferring and adding the incomplete outputs. Thus, this block will be mapped directly, as with a regular block.

#### 3.3.3. Mapping Method for Case 2

**Figure 5:**
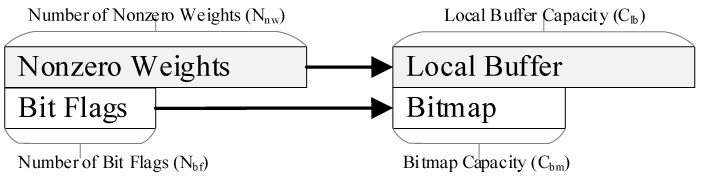
The compressed weights in the local buffer.

**Figure 6:**
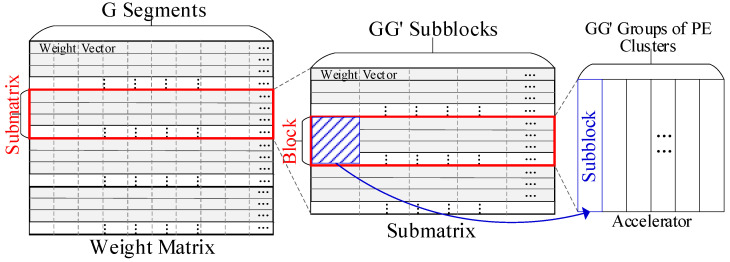
The mapping scheme for irregular case 2.

Weight vectors are stored in local buffers in a compressed format, as shown in Figure 5. The original length of each weight vector is equal to the number of its bit flags, denoted as $N_{bf}$. Let $C_{lb}$ and $C_{bm}$, respectively, denote the capacity of a local buffer and the capacity of its bitmap. Apparently, $C_{bm}$ should be larger than $C_{lb}$ since weights are sparse and compressed. However, when loaded into a PE, a compressed vector may exceed the capacity of either the local buffer or the bitmap. The mapping scheme for the compressed weight matrix should consider the number of both bit flags and nonzero weights, while the latter varies among the vectors. Furthermore, for any vector, all parts of it should be processed together on-chip. Otherwise, its incomplete outputs will be sent to DRAM, which will cause notable DRAM accesses. To address this issue, the mapping method involves two steps, as shown in Figure 6. This method is essential to mapping the compressed weight matrices.

Step 1: Ensure that the bitmap can accommodate the bit flags of each weight vector. First, find the factors that can evenly divide the number of PE clusters, N (e.g., 1, 2, 4, and 8 for N=16), and choose the smallest one that is greater than or equal to $N_{bf}/C_{bm}$, denoted as G. Apparently, if $N_{bf}<C_{bm}$, G will be 1.

Then, split the weight matrix into submatrices with NP/G vectors in each. In this way, the sum of bit flags in each submatrix will not exceed the total capacity of bitmaps in the accelerator. Next, evenly divide each uncompressed weight vector into G segments. Each segment is intended to be stored in a local buffer. As discussed in Case 1, each submatrix can be mapped by dividing the PE array into G groups, with each group consisting of N/G PE clusters.

Now, each PE stores one weight segment from the submatrix. Compress the submatrix and let $N_{nw}$ denote the number of nonzero weights in the longest compressed segment. Apparently, $N_{nw}$ may still exceed the capacity of the local buffer. Hence, the vectors should be further divided.

Step 2: For each submatrix, denote G′ as the smallest factor that evenly divides N/G and is greater than or equal to $N_{nw}/C_{lb}$. Then, partition the submatrix into blocks with $NP/\left(GG\prime \right)$ vectors in each.

The size of each block can be analyzed as follows: Cut each vector into GG′ segments evenly, which will divide each block into GG′ subblocks accordingly. If $C_{bm}\leq 2\;\times \;C_{lb}$, the capacity of the local buffer will be sufficient for each segment, allowing each block to fit within the PE array. As discussed in irregular case 1, the PE array will be split into GG′ groups of PE cluster, and each subblock can be loaded into a group. In this way, each block will be fully processed on-chip without sending any incomplete outputs to the DRAM. Apparently, when both G and G′ are equal to 1, case 2 will be the same as the primary mapping scheme.

If $C_{bm}>2\;\times \;C_{lb}$, there is a possibility that the $N_{nw}$ of a subblock is still greater than $C_{lb}$, and the segments still exceed the capacity of the local buffer. Under this condition, we repeat the operations in the above paragraph on the subblocks until they reach the condition of $N_{nw}\leq C_{lb}$. However, to reduce the on-chip data transfers, it is strongly recommended to set $C_{bm}$ to no greater than $2\;\times \;C_{lb}$. This ratio also aligns with the average sparsity of weights.

The operations in case 2 will only partition the weight matrix into blocks. If G or G′ is not equal to 1, the blocks will enter the irregular case 1, as they will contain fewer than N×P vectors.

#### 3.3.4. Overall Workflow of the Adaptable Mapping Scheme

**Figure 7:**
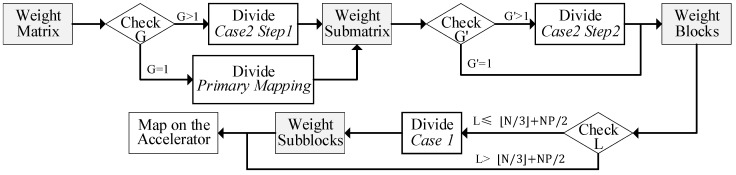
The flowchart of the AMS.

Figure 7 illustrates the flow of the Adaptable Mapping Scheme (AMS). When mapping GEMM on the accelerator, first check G of the weight matrix. If G>1, divide the matrix as depicted in Step 1 of case 2; otherwise, apply the primary mapping scheme. The matrix has been cut into submatrices. Next, check G′. If G′>1, divide the submatrix into blocks as depicted in Step 2 of case 2; otherwise, do not divide the submatrix. Before now, the weight matrix has been divided into blocks, and each block can fit within the accelerator. Afterward, check L for each block. If $L<\left\lfloor N/3\right\rfloor P+NP/2$, divide the block into subblocks as in case 1, and the subblocks within each block will be mapped onto PE groups. Otherwise, each block can be directly mapped.

It should be noted that all division operations on vectors in both case 1 and case 2 are performed based using the uncompressed format. Each segment of the weight vectors within the same division contains an equal number of bit flags. After mapping, bitmaps in each PE will also store an equal number of bit flags. When the PE array stores multiple weight blocks (or subblocks), each local buffer will sequentially store corresponding vectors (or segments).

## 4. Sparse and Balanced Storage System

**Figure 8:**
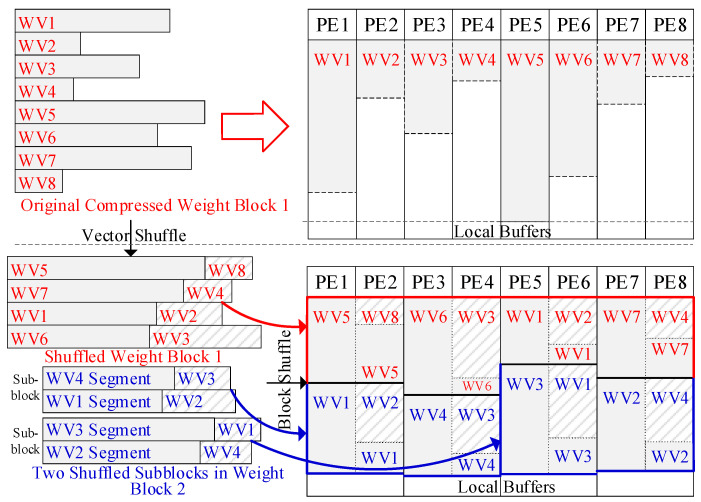
The diagram of the shuffle strategy. With the sparsity-aware shuffle strategy, the PE array can accommodate both Blocks 1 and 2. Block 2 is cut into two subblocks and stored in two PE groups, PE1 to PE4 and PE5 to PE8.

As discussed in Section 3.2, when mapping GEMM onto the accelerator, multiple weight blocks can be loaded into the accelerator simultaneously; each PE will hold multiple vectors, with one vector from each block, provided that the local buffer capacity is large enough. The input vectors in the shared buffer will be reused by all these weight blocks. Since this approach minimizes the repeated DRAM accesses of inputs, it should load as many weight blocks as possible into the accelerator. However, due to the compression, weight vectors have irregular lengths, leading to the imbalanced utilization of local buffers. Figure 8 shows the mapping on eight PEs, where each PE has a 1 KB local buffer and a 2 KB bitmap, assuming each weight is quantized to 1 byte. Initially, Block 1 has eight vectors and each vector has 1 K weights, while Block 2 has four vectors with 2 K weights in each.

When mapped using the AMS in Section 3.3, the local buffer of PE5 is left with minimal space after storing the compressed vector 5 from Block 1, preventing it from storing another vector. Hence, the PE array can only accommodate Block 1, even though the bitmaps and other local buffers have abundant available space. Besides this, irregularity also exists among blocks. Each block shows the distinct distribution of vector lengths. When two blocks are loaded into the PE array together, some PEs may store two long vectors, while other PEs store two short ones from respective blocks. This will worsen the unpredictability and imbalance nature of buffer utilization. When storing more blocks in the PE array, this issue will be more pronounced. The two-level irregularity wastes the space of local buffers and requires repeated DRAM accesses for inputs. Therefore, the weight vectors should be reordered according to their sparsity before loading into the accelerator.

### 4.1. Offline Sparsity-Aware Shuffle Strategy

To achieve the balanced utilization of buffers among both weight vectors and blocks, a two-level sparsity-aware shuffle strategy is applied offline, as shown at the bottom of Figure 8. The shuffle operation is first executed at the vector level. First, we rank the weight vectors in a block based on the number of nonzero weights in each. Then, we pair the vector containing the most nonzero weights with the vector containing the fewest. We repeat this process on the remaining vectors until they all get paired. This will significantly reduce the length differences between the vector pairs. Next, we allow each pair of adjacent PEs to share their local buffers; for example, PE1 and PE2 can access each other’s local buffer. Then, each vector pair is stored in the shared local buffers of a PE pair. For example, WV5 and WV8 from Block 1 are stored together in the local buffers of PE1 and PE2, as shown in Figure 8. This strategy will effectively alleviate the imbalance in buffer utilization caused by the irregular vector lengths. It is also applied on the segments of vectors, such as the segments of Block 2 in Figure 8. It should be noted that the shuffle operation is performed on weight vectors within each block and on segments within each subblock. Each block or subblock should be mapped within one PE group and share the inputs. Cross-group shuffle will disrupt the inputs’ reuse and computing flow.

After the vector shuffle operation, the difference in vector lengths will be reduced. However, imbalances in the buffer utilization may still be not trivial due to the irregular sizes of vector pairs. Therefore, the shuffle operation is also applied at the block level before loading multiple blocks into the PE array.

**Figure 9:**
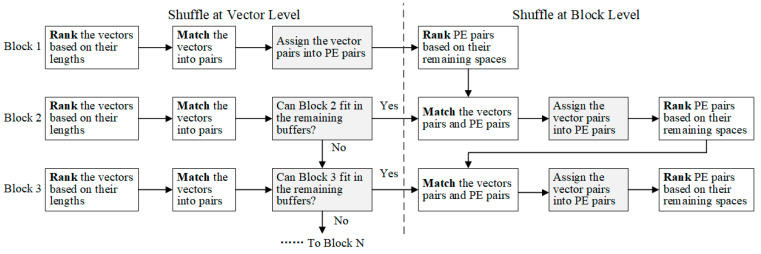
The diagram of two-level sparsity-shuffle strategy.

First, we calculate the remaining buffer spaces for each PE pair after storing the first block, such as Block 1 in Figure 8. Then, we rank the PE pairs based on the remaining buffer spaces, and rank the shuffled vector pairs in the next block (such as Block 2 in Figure 8) by length. Next, we load each vector pair into the PE pair in descending order, placing the longest vector pair in the PE pair with the most available buffer space. Likewise, if the next block has been divided into subblocks, each subblock will be mapped on a corresponding PE group. Then, we rank the segments within each subblock and rank the PE pair within the PE groups. Next, we match the segment pairs with PE pairs in the corresponding PE groups in descending order, as shown with Block 2 in Figure 8. After storing two blocks, we calculate the remaining buffer space and repeat the process for the next block. If the available space in the local buffers is insufficient for the next block (i.e., one or more vector pairs exceed the remaining space after shuffled), then we try another block. If none of the remaining blocks can fit in the local buffers, the PE array is considered full, and the stored blocks are processed with all input vectors. Figure 9 summarizes the process of the two-level sparsity-aware shuffle strategy.

This approach significantly balances the utilization of local buffers, enabling them to accommodate more weight blocks. Each PE’s local buffers will store a similar number of nonzero weights, while each bitmap contains an equal number of bit flags. The outputs will be restored to the original order in the adder tree, which is easily achieved on-chip since the shuffle operation is confined within each PE group. The shared buffer and the storage of inputs will remain unaffected by the shuffle strategy.

### 4.2. The Physical Design of Local Buffers

**Figure 10:**
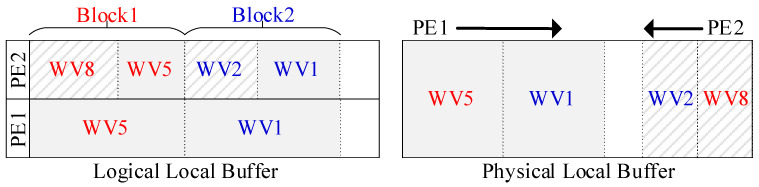
Logical and physical local buffer.

In the shuffle strategy, logically, each set of two vectors or segment pair will be stored in the shared local buffers of two adjacent PEs, as shown on the left side of Figure 10. To adapt to this, physical local buffers will be designed with the dual-port SRAM, instead of single-port SRAM, as shown on the right side of Figure 10. A dual-port SRAM with twice the capacity of the single-port SRAM will serve as two local buffers in two adjacent PEs. One PE will access the buffer sequentially, from top to bottom (e.g., PE1 in Figure 10), while the other will access inversely, from bottom to top (e.g., PE2). Each PE will store its weight vectors separately in the shared local buffer. Thus, each PE can access its related weight vectors. For instance, PE1 can access WV5 and WV1, while PE2 can access WV8 and WV2, as shown in Figure 10. This doubles the addressable logical space in each local buffer with the same physical on-chip memory footprint. Moreover, this design perfectly aligns with the configurable memory modules on the FPGA, such as Block RAM.

### 4.3. On-Chip Data Transfer and Loading Flow

When the accelerator processes GEMM, both weights and inputs should be loaded into buffers before computing. The loading speed must match the data consumption rate of the PE array to avoid stalls during computation.

Each shared buffer can access the DRAM interface independently for inputs. We assume the accelerator contains N PE clusters and each PE cluster contains P PEs, with each PE consisting of one MAC unit. At each cycle, N inputs are transferred to the accelerator at most. This only happens when each PE cluster works as an independent group, which is a very rare case. In all other cases, multiple PE clusters will share the inputs. Moreover, the inputs can be reused by multiple weight blocks over several cycles. Hence, the bandwidth of DRAM access required by inputs is very small—less than N.

Meanwhile, the outputs and weights will time-share the DRAM bus. Each PE cluster can load only one weight per cycle from the DRAM interface, but it processes P weights per cycle, resulting in a severe bandwidth bottleneck. Fortunately, weights are highly reused in the WOS dataflow. Each weight will be computed with I×B inputs, as discussed in Section 3.2. Therefore, the bandwidth bottleneck can be avoided by reusing the weights for multiple cycles with multiple inputs. What’s more, when computing the current weight blocks in local buffers, the accelerator can load a new block in parallel. The new one can directly replace the old one in the local buffer if the old one has been fully computed. The outputs of each PE will be transferred to DRAM when the DRAM bus is free. If the outputs are the inputs for the next GEMM operation, they will be organized to the required format and compressed before storing into DRAM.

Under this method, the accelerator needs a relatively small bandwidth of DRAM interface. It also eliminates the need for long-distance communication between PEs and on-chip buffers. This can significantly reduce the overhead of NoC, and save a lot of energy.

## 5. Sparse Computing Method and Flow

### 5.1. The Sparse Computing Method

On the GEMM accelerator, weights and inputs are computed without decompression to reduce both latency and energy consumption. As mentioned in Section 3.2, each nonzero input is paired with a code to record the number of zeros between it and the preceding nonzero input. Meanwhile, a bitmap is added to each local buffer and only nonzero weights are stored in the local buffer, as shown in Figure 10. A bitmap pointer (BMP) and a local buffer pointer (LBP) are added to the bitmap and local buffer, respectively, in each PE. Each pointer keeps the currently accessed address. The sparse computing method is designed based on these compressed formats, as described below, using one PE cluster for simplicity.

**Figure 11:**
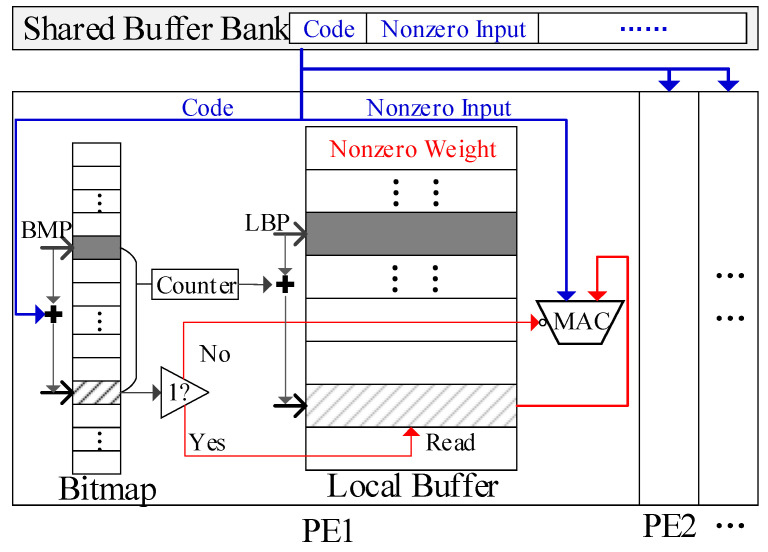
The sparse computing method with the compressed format.

As shown in Figure 11, first, a nonzero input and its code is broadcasted to all PEs. Then, in each PE, the BMP is updated by adding the code to its current value, which will point to a new address in the bitmap. The bit flag at this address marks whether the target weight in this PE is nonzero. Next, a counter tallies the bit flags with a value of 1 between the old and new addresses in the bitmap. This count indicates the number of nonzero weights between the target weight and current weight in the local buffer. Next, we update the LBP by adding the count to its current value. This will point to a new address storing a new nonzero weight in the local buffer. Finally, we check the new bit flag in the bitmap. If it is 1, the new nonzero weight will be the target weight. We read this weight from local buffer and send it to the MAC unit for computation. If the bit flag is 0, then we mask both the buffer access and MAC computing to save energy. The five steps mentioned above are executed in a pipelined manner, allowing five inputs to be processed for different steps synchronously in each PE. Consequently, each PE can handle one nonzero input at each cycle, preventing any computing stalls.

The above method can be generalized to PE groups of any size. In each group, all PEs will share the same inputs, and execute the sparse computing flow independently. When the PE array is split into multiple groups, each group will run the computing flow independently with its own inputs.

This approach can reduce both the computing latency and the energy consumption. The hardware overhead is minimal. No extra circuits are required in the shared buffer. Only two registers, two adders, and one counter are added to each PE. The size of the adders and counters is constrained by the bit width of code for the nonzero inputs, which is 4 bit in this paper, as discussed in Section 3.2.

### 5.2. The Sparse WOS Dataflow

The sparse WOS dataflow begins with mapping the GEMM on the accelerator. Before loading the weight matrix into the PE array, the AMS is applied to divide the matrix into blocks (or subblocks if the blocks are of irregular size). Then, vector-level shuffle operations are performed within each block to generate vector pairs. Next, we execute the block-level shuffle operations on the blocks sequentially. All of these steps are carried out offline. Afterward, the shuffled weight blocks will be loaded into the PE array. The inputs will be stored in the shared buffer, and aligned with the weight blocks in the same PE group. Finally, the sparse computing flow is performed on the compressed inputs and weights in each PE. The outputs will be compressed and stored in DRAM.

## 6. Implementation, Experiments and Discussions

### 6.1. The Implementation of the GEMM Accelerator

The proposed architecture of the GEMM accelerator is validated with FPGA. Before implementation, the capacity of local buffers should be decided based on the specific application requirements first. Other parameters in the implementation, such as the array size, bit width, and shared buffer capacity, have minimal impacts on the proposed architecture. They can be adjusted as required. In this paper, the GEMM accelerator is implemented on a Kintex-7 series FPGA device XC7K325T-2FFG900 from Xilinx, San Jose, CA, USA, with the following parameters.

Total number of PE clusters: 16.

Number of PEs in each cluster: 16.

Bit width of the MAC units: 16 bit.

Precision of the weights and inputs: Int16.

Capacity of each shared buffer bank: 1 KB (16 KB in total).

Capacity of each local buffer: 2 KB (512 KB in total).

Capacity of the bitmap in each PE: 2 KB.

MAC units are designed with the dedicated digital signal processing (DSP) slices (DSP48E1) provided by the Kintex-7 FPGA. Each DSP slice is used as one Int16 MAC unit. Both shared buffers and local buffers are implemented with the Block RAMs (BRAMs), while the bitmaps are stored in the distributed RAMs. The proposed GEMM accelerator is implemented on the Genesys 2 board from Digilent, Pullman, WA, USA, which features the XC7K325T-2FFG900C FPGA and DDR3 memory. The Zybo Z7 SoC development board, equipped with the Zynq-7000 ARM/FPGA SoC, serves as the external inspector for collecting experimental results.

### 6.2. The Tested Samples of GEMM

To validate the proposed accelerator, it will be tested with GEMM operations from multiple neural networks, including the fully-connected (FC) layers of CNNs (VGG16, ResNet50, MobileNetv3), the gating mechanism of LSTM, and the multi-head self-attention (MHSA) and feedforward network (FFN) of the transformer. As with other works, Giga Operations per Second (GOPS) is used to measure the computing performance of the accelerator, where each MAC computation counts as two operations. GOPS/W indicates the energy efficiency, representing the computing performance created per watt. GOPS is calculated as follows:

(1) $$GOPS=\frac{Total\;Number\;of\;\;Computing\;Operations\;in\;the\;GEMM\;}{Total\;Number\;of\;Seconds\;for\;Processing\;the\;GEMM}$$

where all operations are quantized to 16-bit in this paper for comparison. Table 1 lists the tested samples for GEMM and their dimensions. These test samples covered CNN, RNN and transformer. They are retrained with Int16 in this work. The sparsity of each of the weight vectors in those samples ranges from 12% to 83%.

### 6.3. The Experiments Setup of Proposed GEMM

The proposed GEMM accelerator is implemented with Verilog HDL on the Vivado Design Suite 2022.1 and then programmed onto the XC7K325T FPGA. The samples in Table 1 will be processed on the proposed GEMM accelerator and the inspector on the Zybo Z7 board will count the processing cycles for each sample. The power performance of the proposed accelerator is evaluated with Xilinx FPGA and SOC design suite, Vivado 2022.1. The results for running power are obtained after the synthesis and implementation of the proposed accelerator, with the testbenches of GEMM samples in Table 1. The energy consumption of the implemented accelerator will be estimated using the Switching Activity Interchange Format (SAIF) files on Vivado.

This paper proposes an accelerator based on the WOS dataflow architecture and introduces three optimization techniques for GEMM operations, as follows: an adaptable mapping scheme (AMS), a sparsity-aware offline shuffle strategy, and a sparse computing method. The efficiency and effectiveness of the proposed accelerator are verified through testing. First, the WOS dataflow is evaluated and compared with the weight-stationary (WS) and output-stationary (OS) dataflow baselines. Then, the complete GEMM accelerator, incorporating the proposed techniques, is verified and compared with existing works.

### 6.4. The Validation of WOS Dataflow

The primary architecture of the GEMM accelerator in Section 3.1 is implemented on FPGA first to verify the WOS dataflow without any additional techniques. The WS and OS accelerator are also implemented as baselines in the systolic architecture, which refer to TPU [15] and Thinker [16], respectively. A PE array of size 16 × 16 is equipped in all the baselines. The capacity of buffers in each baseline is set as follows:

OS—16 KB weight buffer and 512 KB input buffer;

WS—16 KB weight buffer, 256 KB input buffer, and 256 KB output buffer (for partial sums).

**Figure 12:**
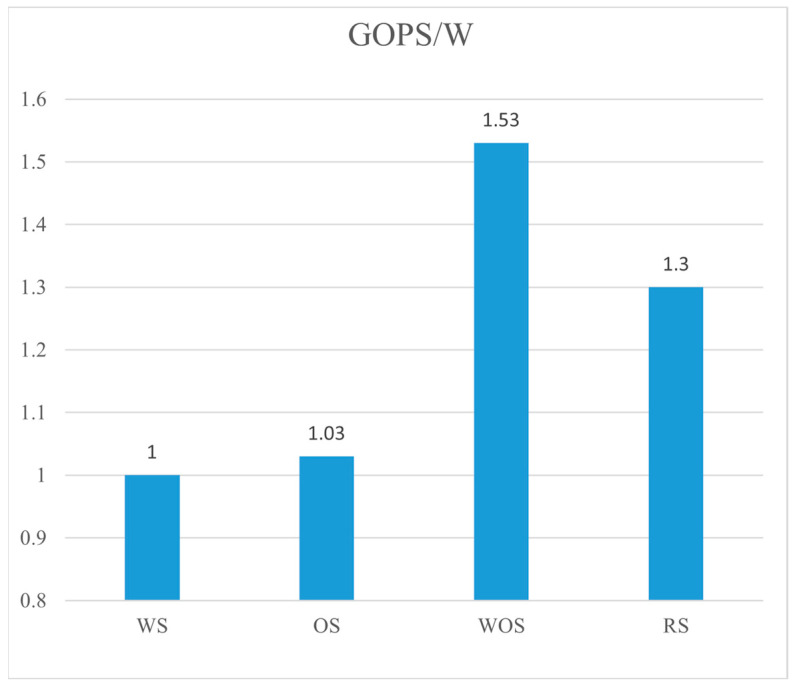
The energy efficiency of different dataflows. All GOPS/W is normalized to the WS dataflow.

All dataflow accelerators are tested with samples MHSA1, MHSA2, FFN1 and FFN2 in Table 1. These samples achieve the full utilization of PEs, which isolates the efficiency of WOS dataflow. Figure 12 compares the GOPS/W across different dataflows. RS refers to the row-stationary dataflow in Eyeriss, which reports 1.3× better energy efficiency than WS and OS dataflow. The proposed WOS dataflow achieves the highest GOPS/W, which is 1.53× higher than the WS dataflow. This improvement is attributed to the significant reduction in on-chip data transfers in WOS dataflow. In WOS dataflow, only input data need to be transferred across the PE array during GEMM processing, whereas OS and WS dataflows require additional transfers of weights and partial sums, respectively. Compared with the proposed WOS dataflow, RS dataflow in Eyeriss relies on a more complex NoC, including a global Y bus, a global X bus, and a multicast controller for each PE and X bus to decode the ID (column and row) of each point of data. Additionally, it suffers from on-chip data duplication.

The DRAM accesses of OS, WS and WOS dataflows are also evaluated. When processing the GEMM with batch mode in Table 1, the average DRAM accesses for the proposed WOS dataflow are 82% fewer than those of the WS dataflow, and nearly equivalent to those of OS dataflow. Work [12] reports that the RS and OS dataflows have similar amounts of DRAM accesses.

### 6.5. The Validation of the Complete GEMM Accelerator

To verify the proposed GEMM accelerator, it will process all the samples listed in Table 1. The total number of MAC computations of each sample is obtained by multiplying the batch size, the number of rows in the input matrix and the size of the weight matrix. The inspector on the Zybo Z7 board will count the processing cycles for each sample.

#### 6.5.1. The PE Utilization of GEMM Accelerator

Firstly, PE utilization will be evaluated. It is calculated as follows:

(2) $$PE\;Util=\frac{Total\;Number\;of\;MAC\;Computation}{Total\;Computing\;Cycles\times Number\;of\;PEs},$$

which indicates the utilization of computing resources and demonstrates how many PEs are assigned with workloads when processing a GEMM operation. Idle PEs will be wasted. Thus, this metric can reveal the applicability of the proposed AMS for GEMM with various sizes. In this experiment, each PE contains one DSP. The experiment results show that the GEMM accelerator achieves an average PE utilization of 95.66%, which is exceptionally high compared to the other results in current works, such as 84.8% reported in Thinker [16]. The slight loss in PE utilization is due to the imbalanced computing loads among PE groups, which only occurs in the irregular weight blocks. The results testify that the AMS is very effective for the compressed GEMM operations in major neural networks.

#### 6.5.2. The Resource Utilization of the Proposed GEMM Accelerator

Table 2 shows the experimental results of the proposed accelerator and a comparison with current works, including the resources utilization, computing efficiency and energy efficiency.

An efficient GEMM accelerator should minimize hardware resource consumption to reduce hardware overhead and save energy. Table 2 presents the utilization of lookup-tables (LUTs), BRAMs, flip-flops (FFs) and DSPs for the proposed GEMM accelerator, alongside comparisons with other works. It shows that the proposed accelerator consumes significantly fewer resources than most compared works. This is mainly attributed to the fact that most of the techniques proposed in this work are operated offline, including the adaptable mapping scheme and the sparsity-aware shuffle strategy. Besides this, the proposed accelerator also benefits from the low-cost designs, including the simplified NoC, the shared controlling logics, and the sparse processing method based on the indirect address. Some works report lower resource utilization, but they do not exploit the sparsity of GEMM.

Meanwhile, DSPs are used as the primary computational resources, with all other resources acting as auxiliary. Therefore, the amount of LUTs and FFs consumed per DSP can serve as an indicator of the accelerator’s hardware overhead. The proposed accelerator uses fewer LUTs and FFs per DSP compared to most current designs, further demonstrating its efficiency in resource utilization.

#### 6.5.3. The Computing Efficiency of the Proposed GEMM Accelerator

GOPS indicates the computing performance of each accelerator. Table 2 shows that the proposed accelerator achieves a GOPS of 196.08, which is relatively higher than those in other works. Furthermore, since the computing performance is proportional to both the number of DSPs and the operating frequency, a normalized parameter, GOPS/DSP/F, is introduced to reflect the computing efficiency of the accelerator. This parameter is calculated as follows:

(3) $$GOPS/DSP/F=\frac{GOPS}{Total\;Number\;of\;DSPs\times Frequency},$$

which is presented in the 10th column of Table 2. It can clearly demonstrate the computing performance that each DSP achieves per unit of frequency. Table 2 shows that the proposed GEMM accelerator achieves the highest computing efficiency. Its GOPS/DSP/F is at least 1.73× higher than those in other works. Apparently, when implemented with the same number of DSPs and operating at the same frequency, the proposed accelerator will achieve significantly higher computing performance compared to other works.

Two factors can explain the high computing efficiency of this work. Firstly, the proposed accelerator fully utilizes the DSPs. As evaluated in the above paragraphs, it achieves a high PE utilization of 95.66% due to its AMS technique. This advantage significantly improves the average computing throughput of each DSP. Secondly, the sparse computing method skips the MAC computation of all zeros in the input matrices, which will remarkably reduce the computing latency. As illustrated in Equation (1), this will greatly improve the effective GOPS of the accelerator. Consequently, the proposed accelerator can obtain a high computing efficiency.

#### 6.5.4. The Energy Efficiency of the Proposed GEMM Accelerator

Table 2 also shows the energy performance of the proposed accelerator compared to other designs. Since the power consumption is closely related to the hardware resource utilization and operating frequency, most works adopt GOPS/W to measure the energy efficiency. As shown in the 11th column of Table 2, the proposed accelerator achieves the highest energy efficiency, which is at least 1.36× higher than those of other works. Furthermore, it is 5× more energy efficient than NVIDIA Tegra x1 GPU, the energy efficiency of which is reported to be 6 GOPS/W in NullHop [19]. This indicates that the proposed accelerator offers more computing throughput with the same energy consumption.

The high energy efficiency is attributed to the architecture of the proposed accelerator. Firstly, the proposed architecture features a low-cost design. As discussed above, the proposed accelerator utilizes relatively fewer hardware resources, resulting in lower power dissipation, especially regarding static power. Secondly, the proposed accelerator also improves the energy efficiency with its sparse computing flow. By skipping the computations with zeros in the inputs, the sparse computing method can completely remove all the energy consumption for them, including computing, storage and transfer. Meanwhile, when computing the weights with zero values, the DSPs will be disabled, leading to substantial energy savings. Moreover, the sparse dataflow will also reduce the energy consumption by eliminating the transfer and storage of zeros in weights. The thorough exploitation of the sparsity in GEMM operations explains why the proposed accelerator can outperform other sparse accelerators, such as NullHop [19], in terms of energy efficiency. Finally, the idle PEs with no computing workload waste notable energy, without contributing to the computing throughput. The proposed accelerator achieves high PE utilization, which can minimize the energy waste. All these factors make the proposed architecture much more energy efficient.

### 6.6. Discussion and Summary

The accelerator in this paper is designed to process the GEMM operations with low-cost, low energy consumption and real-time speed for resource-constrained applications. The key focus is on improving both computing and energy efficiency, which means offering more computing throughput than other designs with the same computing resources and energy consumption. The experimental results show that the proposed accelerator achieves superior computing and energy efficiency, primarily through increased resource utilization and reduced energy consumption. The proposed accelerator is also evaluated with two other PE array sizes: one with eight PE clusters, each containing 16 PEs, and another with eight PE clusters, each containing 16 PEs. The experimental results show that they achieve similar GOPS/DSP/F and GOPS/W as the implementation in Table 2.

Firstly, this approach leverages the WOS dataflow as the principal architecture. WOS dataflow can keep both weights and partial sums stationary in the PEs during processing, thereby reducing energy consumption for data transfers. Secondly, the AMS is introduced to improve the PE utilization for GEMM with varying matrix sizes. It achieves an average PE utilization of 95.66%. This significantly enhances the utilization efficiency of computing resources and boosts overall computing performance. Thirdly, the accelerator also adopts the sparsity-aware offline shuffle strategy to manage the irregular lengths of compressed weight vectors. This strategy can greatly balance the utilization of local buffers. The buffer utilization is increased, allowing the accelerator to store more weight blocks. As a result, it increases input reuse and reduces repetitive access to DRAM, contributing to energy savings. Finally, the accelerator also benefits from the sparse processing flow. It achieves a high effective GOPS by skipping computations with zeros in the inputs. Besides this, it saves energy by omitting the on-chip transfer and storage of zeros in the weights and disabling their computation.

Therefore, the architecture and techniques are very efficient in terms of energy and computation. They are not limited to a specific implementation. The accelerator can be deployed with varying sizes of PE arrays or operated at different frequencies, while still maintaining high efficiency.

## 7. Conclusions

This paper proposes and implements an architecture for GEMM accelerator, featuring a low hardware overhead, high computing throughput and low energy consumption. It is designed based on the WOS dataflow, incorporating the AMS, the sparsity-aware shuffle strategy and the sparse computing method. By processing the GEMM in compressed format, it achieves high energy and computing efficiency. This architecture is presented for resource-constrained applications. Yet experiments imply that this architecture can still consume less energy and achieve high computing performance with a large PE array and high operating frequency. Thus, this paper’s approach can also be extended to the large-scale processing units for GEMM.

## Figures and Tables

**Table 1:** The tested samples of GEMM.

GEMM	Batch Size	Dimension		Overall Sparsity
		Input Matrix	Weight Matrix	
VGG16	16	1×4096	4096×1000	42.37%
ResNet18	16	1×512	512×1000	36.58%
ResNet50	16	1×2048	2048×1000	40.85%
MobileNet-v3-Small	16	1×576	576×1280	56.38%
	16	1×1280	1280×1000	63.22%
Forget Gate	32	1×1024	1024×768	48.62%
Input Gate	32	1×1024	1024×768	43.27%
Output Gate	32	1×1024	1024×768	45.62%
MHSA1 ^1^	4	32×512	512×64×8	39.77%
MHSA2 ^2^	4	32×512	512×512	42.18%
FFN1	4	32×512	512×2048	33.79%
FFN2	4	32×2048	2048×512	42.55%

^1^ Calculate the queries, keys and values with weight matrices in eight heads. ^2^ Calculate the multi-head attention with weight matrices.

**Table 2:** The experimental results of the proposed GEMM accelerator and the comparison with existing works.

Works	Device	LUT	FF	BRAM ^1^	DSP ^2^	Freq (MHz)	Power(W)	GOPS	GOPS/DSP/F ^4^	GOPS/W	Year
[29]	Cyclone V DE1-SoC	25,281	/	234 KB	136 ^3^	140.9	14.2	35.72	1.86	2.52	2023
[31]	Alveo U250	736K	/	1781	4189	300	77.17	1800	1.43	23.33	2023
[30]	XCVU19P	248,239	/	320 KB	128	100	2.14	25.6	2.00	11.96	2022
[2]	XC7VX690T	468K	649K	1465	1436	200	17.3	433.63	1.51	25.06	2019
[19]	Zynq-7100	229K	107K	386	128	60	0.8	17.19	2.24	21.49	2019
[45]	Arria 10 GX	1380K	966K	3800 KB	726	236	18	156.8	0.92	8.71	2024
[46]	Alveo U280	623K	793K	1163	2251	200	43.7	238.2	0.53	5.45	2023
This work	XC7K325T	83,165	41,276	134	256	200	5.83	196.08	3.87	33.98	2024

^1^ Each BRAM is a 4 KB SRAM. This column reports the number of used BRAMs or the on-chip buffer capacity. ^2^ Each DSP can be used as one 16 × 16 MAC unit, such as the DSP48E1 slices. ^3^ It utilizes 68 DSP blocks, and each can be used as two 16 × 16 MAC units. ^4^ F is short for frequency in the 7th column and it is calculated with GHz in the 10th column.

## Data Availability

The raw data supporting the conclusions of this article will be made available by the authors on request.
